# Large Language Model-Based Automated Tumor, Node, Metastasis Staging and Resectability Assessment for Pancreatic Cancer in Radiology Reports With Detection of Incomplete Documentation

**DOI:** 10.7759/cureus.96448

**Published:** 2025-11-09

**Authors:** Shota Fujisawa, Ryo Kurokawa, Akifumi Hagiwara, Mariko Kurokawa, Yusuke Asari, Sousuke Hatano, Daisuke Matsumoto, Ako Negishi, Wataru Gonoi, Osamu Abe

**Affiliations:** 1 Radiology, The University of Tokyo, Tokyo, JPN; 2 Radiology, Juntendo University, Tokyo, JPN

**Keywords:** claude 3.7 sonnet, ductal pancreatic carcinoma, large language models, radiology reports, resectability, tnm stage

## Abstract

Objectives: To assess the performance of the large language model (LLM) Claude 3.7 Sonnet in automatically extracting tumor, node, metastasis (TNM) classification and resectability status, as well as in identifying missing information required for these assessments, from free-text Japanese radiology reports of pancreatic cancer.

Methods: We conducted a retrospective study of 101 Japanese radiology reports from pancreatic cancer staging computed tomography (CT) examinations. Using a zero-shot approach, Claude 3.7 Sonnet was prompted with definitions from the 8th edition of the Japanese Pancreatic Cancer Classification to generate TNM stage and resectability categories. The model’s outputs were compared with a reference standard established by a radiologist’s interpretation of the same reports. Performance metrics included categorical accuracy and Cohen’s kappa coefficients. Detailed error analysis was also performed to characterize common sources of misclassification.

Results: Claude 3.7 Sonnet achieved accuracies of 84.1% for the T category, 92.1% for the N category, 98.0% for the M category, and 87.1% for resectability. Cohen’s kappa values indicated substantial agreement for T (κ = 0.745) and almost perfect agreement for N (κ = 0.858), M (κ = 0.956), and resectability (κ = 0.812). The lower accuracy in T classification was mainly attributable to misinterpretation of nuanced vascular involvement. The model effectively detected missing information for TNM classification but showed limitations in identifying omissions relevant to resectability assessment.

Conclusion: Claude 3.7 Sonnet demonstrated high accuracy in extracting structured pancreatic cancer staging information from unstructured Japanese radiology reports without task-specific training. While challenges remain in interpreting nuanced descriptions of vascular invasion and resectability, the model reliably identified most staging elements and omissions. These findings highlight the potential of LLMs as tools for semi-automated generation of structured data from routine free-text reports, which could improve reporting consistency, workflow efficiency, and secondary data utilization in oncology care.

## Introduction

Pancreatic ductal adenocarcinoma (PDAC) is among the most lethal malignancies, with five-year survival rates of only around 13% despite advancements in treatment [1]. Early and accurate staging of pancreatic cancer is crucial for guiding therapy and prognosis. In particular, the tumor-node-metastasis (TNM) classification and assessment of resectability status determine the eligibility for surgical resection, the only potentially curative treatment. Radiologists play a key role in staging by interpreting cross-sectional imaging (typically contrast-enhanced computed tomography (CT)) and evaluating vascular involvement to categorize tumors as resectable, borderline resectable, or unresectable. However, conveying this complex information in radiology reports is challenging. Free-text radiology reports often do not explicitly include structured TNM stage or resectability category, leading to underreporting of critical staging details. For example, one survey found that only about 24.5% of radiologists routinely incorporate TNM staging into head and neck cancer imaging reports [2]. In another study of non-small cell lung cancer positron emission tomography/CT (PET/CT) reports, the TNM stage was explicitly stated in just 6% of unstructured text reports, and 22% of reports had incomplete staging information (most often missing T classification) [3]. These findings highlight that a gap in communication-important staging elements may be omitted or difficult to extract from narrative reports - potentially impacting downstream treatment decisions.

In recent years, artificial intelligence (AI) technologies have seen remarkable progress, with growing expectations for their application in various domains of healthcare [4]. Among these, radiology has emerged as one of the most promising fields for AI integration, owing to its inherently data-rich and image-based nature. AI-based tools have been increasingly utilized to support radiologists by automating image analysis tasks, such as tumor detection through advanced image processing techniques. In parallel, large language models (LLMs) have shown potential in assisting with the generation of radiology reports, thereby aiming to reduce the workload of radiologists and improve efficiency [5]. Natural language processing (NLP) approaches have been promising in analyzing free-text medical reports. Traditional rule-based or keyword-based NLP methods can identify certain phrases but struggle with the variability and complexity of radiological language. Recent advances in machine learning, particularly transformer-based language models, have substantially improved information extraction from clinical text. Bidirectional Encoder Representations from Transformers (BERT) and related models have achieved high accuracy in radiology report comprehension tasks. For instance, a German-language BERT model fine-tuned on radiology data achieved an F1 score of ~84% in answering standardized questions from radiology reports and could detect absent (unanswerable) information with 96% accuracy [6]. Transformer models have also been applied to automate cancer staging: a BERT-based classifier trained on text reports attained near-expert performance in determining lung cancer TNM stages from PET/CT radiology reports, correctly classifying all ‘T’, ‘N’, ‘M’, and ‘u’(uncertainty) components in 79% of cases in internal dataset and 84% in external dataset with 0.77-0.95 F1-scores for each component [7]. These results suggest that modern NLP models can reliably interpret nuanced radiological descriptions when appropriately trained.

More recently, LLMs such as the Generative Pre-trained Transformer (GPT) and others (often referred to as “ChatGPT” in the context of conversational AI) have shown remarkable zero-shot and few-shot learning capabilities on medical text without task-specific training [8-14]. In the context of radiology, LLMs have been used to extract information and even generate structured reports from free text. For instance, GPT-3.5 has achieved an accuracy of 69% on multiple-choice questions designed to follow the Canadian Royal College and American Board of Radiology examinations without radiology-specific pretraining [9]. Similarly, GPT-4 has been reported to attain an accuracy of 65% on the Japanese radiology board examination, a performance that closely approaches the official passing threshold [10]. One study showed that Claude 3.5 Sonnet could improve accuracy in Radiology’s Diagnosis Please cases using a two-step approach, in which LLM summarized clinical information and then provided a diagnosis [11]. Another study reported that GPT-4 could transform free-text PDAC CT reports into highly accurate synoptic (structured) reports and correctly categorize tumor resectability when guided with advanced prompting techniques [12]. Another investigation demonstrated that GPT-3.5 could be prompted to output TNM stage from lung cancer CT reports in both English and Japanese, improving accuracy when provided with comprehensive TNM definitions in the prompt [13]. These early applications illustrate the potential of LLMs to serve as powerful clinical text understanding tools. In the domain of pancreatic cancer, Suzuki et al. recently performed a preliminary assessment of GPT-4 on Japanese radiology reports and found reasonable agreement with radiologists’ TNM classification, though performance for T (tumor extent) was notably lower (accuracy ~73%, κ ~0.45) compared to N and M [14]. This underscores that, while LLMs are “familiar” with staging concepts, certain nuances (e.g., distinguishing vascular abutment from invasion for T categorization) remain challenging.

In Japan, standardized criteria for pancreatic cancer staging are defined by the Japan Pancreas Society’s classification system, now in its 8th edition [15]. These guidelines define TNM and resectability classifications that are largely aligned with the National Comprehensive Cancer Network (NCCN) criteria, but differ in some aspects, such as defining T3 as extrapancreatic invasion. Incorporating such definitions into an LLM’s prompt could enable the model to classify findings according to the Japanese system. To date, no study has focused on using LLMs to extract both TNM classification and resectability status from Japanese-language radiology reports of pancreatic cancer using the latest Japanese classification criteria.

In this study, we investigated the performance of a state-of-the-art LLM (Anthropic Claude 3.7 “Sonnet”) in extracting structured TNM stage and resectability category from unstructured Japanese radiology reports for pancreatic cancer. We hypothesized that, with an appropriate prompt aligned to the 8th edition Japanese Pancreatic Cancer Classification, the LLM could accurately reproduce the staging assessments that radiologists imply in their free-text reports. We evaluated the model’s accuracy and inter-rater agreement with radiologist-derived gold standard classifications, and we analyzed common errors to identify where the model may be misinterpreting the reports. We also performed a secondary analysis to assess whether the model could correctly identify missing information (i.e., when certain staging elements were not mentioned in the report). Our goal is to explore the feasibility of semi-automated, AI-assisted generation of structured staging information from routine radiology reports, which could improve clinical data capture and potentially the decision-making process for pancreatic cancer patients.

## Materials and methods

Patient selection and data collection

This retrospective study was approved by the institutional review board of the University of Tokyo Hospital, with a waiver of informed consent due to the use of de-identified data. At our institution, a total of 1,187 CT examinations were performed using a pancreatic dynamic protocol between April 1, 2021, and March 11, 2025. From this initial cohort, 431 scans were excluded as they were performed for pancreatic diseases other than pancreatic cancer. An additional 385 scans were excluded because their scan range did not entirely cover the chest-to-pelvis region. Furthermore, 270 scans representing follow-up examinations (e.g., for assessing chemotherapy response or post-operative surveillance) were also excluded. Consequently, a final cohort of 101 cases was included in the analysis.

All radiology reports were authored in Japanese by board-certified radiologists and documented findings related to the primary pancreatic tumor, regional lymph nodes, and distant metastases as per usual practice. The free-text reports typically also included comments on vascular involvement (e.g., abutment or encasement of arteries/veins), which related to resectability. The reports were exported from the hospital Picture Archiving and Communication System (PACS) in text format, and all protected health information (e.g., patient name, ID, dates) was removed to ensure anonymization. No patient clinical data was provided to the AI. The final dataset for analysis consisted of 101 de-identified reports, each corresponding to one patient’s pancreatic cancer staging CT.

Claude 3.7 prompting strategy

We utilized the Anthropic Claude 3.7 Sonnet, a state-of-the-art large language model, to process each radiology report. The model was accessed via the Anthropic Application Programming Interface (API). We designed a structured prompt to instruct the model to extract the required staging information according to the 8th edition Japanese Pancreatic Cancer Classification [15]. Claude 3.7 was also instructed to list the missing information required to determine the classification for each of the T, N, M, and resectability. The prompt (in Japanese) provided the model with definitions of each TNM category and resectability class, and asked it to read the given radiology report and output a summary of: T category (T1-T4), N category (N0-N1), M category (M0-M1), and resectability status (Resectable (R), Borderline Resectable - Portal Vein involvement (BR-PV), Borderline Resectable - Arterial involvement (BR-A), UnResectable - Locally Advanced (UR-LA), UnResectable - Metastatic (UR-M)). The prompt explicitly instructed the model to adhere strictly to the given definitions and to output the results in a structured JavaScript Object Notation (JSON) format for consistency.

**Figure 1:**
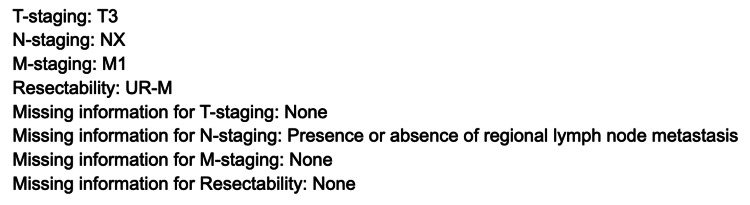
An example of a response generated by Claude T: Tumor; N: Node; M: Metastasis; UR-M: UnResectable - Metastatic

The protocol is as follows(translated into English): 'You are a pancreatic radiologist. Based on the report’s findings, determine: T category, N category, M category, and resectability (Resectable, Borderline, Unresectable) according to the Japanese Pancreatic Cancer Classification 8th edition. Provide the answer in JSON with fields T, N, M, Resectability, and Note (for any uncertainty).' The prompt was concluded with the detailed definitions of T, N, M, and resectability from the Japanese Pancreatic Cancer Classification, 8th edition. In this study, we adopted a zero-shot learning approach. We set the model’s generation temperature to 0 (low creativity), top-p to 1 (the default), and maximum tokens to 1,000 to minimize randomness, aiming for deterministic outputs given the same prompt. A Python script was used to automate the process: it supplied each report to Claude via API call with the fixed prompt and captured the model’s response. Figure 1 shows an example response from Claude.

Reference standard and interpretation

For gold-standard comparison, a single radiologist with four years of experience performed TNM staging (TX, T0, Tis, T1a, T1b, T1c, T2, T3, T4, NX, N0, N1, M0, M1) and assessed resectability status (R, BR-PV, BR-A, UR-LA, UR-M) based solely on the radiological reports (not the images themselves, to simulate real-world usage of reports for data mining). When sub-classifying N1 into N1a and N1b, which requires information on the number of metastatic lymph nodes, the absence of this count in the report was identified as missing information. 

Evaluation metrics

We evaluated the performance of the LLM extraction in two main ways: categorical accuracy for each field and inter-rater agreement with the gold standard using Cohen’s kappa coefficient. For each of the four outputs (T, N, M, resectability), accuracy was calculated as the percentage of cases where the model’s output exactly matched the gold-standard classification. Cohen’s kappa statistic (κ) was computed between the model and the reference standard for each category to account for chance agreement (particularly relevant if class distributions are imbalanced). Kappa was interpreted in the usual way (values >0.8 as almost perfect, 0.6-0.8 as substantial, 0.4-0.6 moderate, <0.4 as fair to poor agreement).

In addition to per-category performance, we assessed the model’s ability to correctly identify when information was missing or indeterminate in the report. If the model output 'unknown' for a category that the radiologists also could not determine from the text (for example, if distant metastases were not commented on at all in the report, both gold standard and model might label M as 'not mentioned,' which we treated as M0 in practice but noted as implicit), this was counted as a correct recognition of missing information. We calculated the missing information detection rate as the proportion of such cases where the model’s handling of an absent finding matched the radiologists’ interpretation (e.g., correctly leaving a category as unspecified when the report provided no evidence). We used scikit-learn (v1.6.1) and statsmodels (v0.14.4) in Python for all statistical calculations (accuracy, κ, etc.). Ninety-five percent confidence intervals for accuracy were obtained by the Wilson score method, and for kappa via bootstrap resampling (10,000 iterations).

## Results

Patient characteristics

The study cohort included 101 patients (53 men and 48 women) with pancreatic cancer. The median age was 72 years (range: 44-93 years). The distribution of patients according to TNM staging and resectability classification is detailed in Table 1. Only 13 patients (12.9%) had tumors confined to the pancreas (T2 stage or lower). Distant metastases were observed in approximately one-third of the cohort (33 patients, 32.7%).

**Table 1:** Distribution of TNM and Resectability Classifications (n = 101) TNM: Tumor, Node, Metastasis; R: Resectable; BR-PV: Borderline Resectable - Portal Vein involvement; BR-A: Borderline Resectable - Arterial involvement; UR-LA: UnResectable - Locally Advanced; UR-M: UnResectable - Metastatic

Category	Number of Patients, n (%)
T Stage	
T1b	1 (1.0%)
T1c	7 (6.9%)
T2	5 (5.0%)
T3	60 (59.4%)
T4	24 (23.8%)
TX	4 (4.0%)
N Stage	
N0	63 (62.4%)
N1	22 (21.8%)
NX	16 (15.8%)
M Stage	
M0	68 (67.3%)
M1	33 (32.7%)
Resectability	
R	47 (46.5%)
BR-PV	4 (4.0%)
BR-A	3 (3.0%)
UR-LA	11 (10.9%)
UR-M	33 (32.7%)
Unclassifiable	3 (3.0%)

Performance of the LLM in extracting TNM and resectability

The large language model Claude 3.7 successfully processed all 101 reports and output TNM classifications and resectability status for each case. Tables 2-5 show the correspondence between the determinations (or classifications) made by Claude and those made by the physician. In brief, the model’s output matched the expert reference standard in 84.1% (95% confidence interval (CI): 75.8%-90.0%) of cases for the T category, 92.1% (95%CI: 85.1-96.0%) for N category, 98.0% (95%CI: 93.1-99.5%) for M category, and 87.1% (95%CI: 79.2-92.3%) for overall resectability status. The corresponding Cohen’s kappa values were κ = 0.745 (95%CI: 0.622-0.849) for T (substantial agreement), κ = 0.858 (95%CI: 0.755-0.946) for N (almost perfect agreement), κ = 0.956 (95%CI: 0.885-1.00) for M (almost perfect agreement), and κ = 0.812 (95%CI: 0.716-0.898) for resectability (almost perfect agreement).

**Table 2:** Correspondence Between T Category Classifications by Claude and the Radiologist T: Tumor

Claude Classification	Radiologist Classification					
	T1b (Radiologist)	T1c (Radiologist)	T2 (Radiologist)	T3 (Radiologist)	T4 (Radiologist)	TX (Radiologist)
T1b(Claude)	1	0	0	0	0	0
T1c(Claude)	0	7	0	1	0	0
T2(Claude)	0	0	5	5	0	0
T3(Claude)	0	0	0	49	3	1
T4(Claude)	0	0	0	3	20	0
TX(Claude)	0	0	0	2	1	3

**Table 3:** Correspondence Between N Category Classifications by Claude and the Radiologist N: Node

Claude Classification	Radiologist Classification		
	N0 (Radiologist)	N1 (Radiologist)	NX (Radiologist)
N0 (Claude)	57	0	1
N1 (Claude)	4	22	1
NX (Claude)	2	0	14

**Table 4:** Correspondence Between M Category Classifications by Claude and the Radiologist M: Metastasis

Claude Classification	Radiologist Classification	
	M0 (Radiologist)	M1 (Radiologist)
M0 (Claude)	66	0
M1 (Claude)	2	33

**Table 5:** Correspondence Between Resectability Classifications by Claude and the Radiologist R: Resectable; BR-PV: Borderline Resectable - Portal Vein involvement; BR-A: Borderline Resectable - Arterial involvement; UR-LA: UnResectable - Locally Advanced; UR-M: UnResectable - Metastatic

Claude Classification	Radiologist Classification					
	R (Radiologist)	BR-PV (Radiologist)	BR-A (Radiologist)	UR-LA (Radiologist)	UR-M (Radiologist)	Unclassifiable (Radiologist)
R (Claude)	39	0	0	0	0	0
BR-PV (Claude)	0	3	0	0	0	0
BR-A (Claude)	3	0	3	1	0	0
UR-LA (Claude)	3	1	0	10	0	3
UR-M (Claude)	2	0	0	0	33	0

Error analysis

The physician identified informational deficiencies in five cases for the T-factor, 35 cases for the N-factor, two cases for the M-factor, and six cases for resectability classification. The rates at which Claude correctly identified these same physician-flagged omissions were 100% for the T-factor, 85.7% for the N-factor, 100% for the M-factor, and 50% for resectability classification.

## Discussion

This study demonstrated that, without task-specific training, Claude 3.7 Sonnet can determine TNM staging and resectability for pancreatic cancer from radiology reports with high overall accuracy, though T-staging accuracy was lower. While its ability to identify missing information was successful for TNM classification, it was limited for resectability. These findings are consistent with previous research, including an LLM-based analysis of Japanese pancreatic cancer reports [14] and a study on resectability classification from structured reports [16]. However, our accuracy was lower than the 92% achieved in a study that employed a Chain-of-Thought technique with few-shot learning to classify resectability [12]. This disparity indicates that, for complex tasks such as resectability classification, the integration of the Chain-of-Thought technique into the prompt may be a key factor for enhancing accuracy.

The primary strength of this study lies in its detailed qualitative error analysis. Rather than merely reporting accuracy metrics, we delved into the specific reasons for misclassifications by the LLM. By identifying the model's struggles with nuanced clinical language (e.g., 'contact' vs. 'invasion'), complex peri-pancreatic anatomy, and quantitative assessments such as the degree of vessel involvement, we provide crucial insights into the current limitations of LLMs in radiology. These findings are essential for guiding future model development and for establishing best practices for human-AI collaboration.

The relatively low accuracy in T-staging was primarily due to the misclassification between T3 and T4 stages and the under-staging of advanced tumors. These errors suggest the model struggled to interpret nuanced descriptions of vascular involvement (e.g., failing to recognize that vascular 'contact' is a significant indicator of 'invasion') and lacked a full understanding of peri-pancreatic anatomy, such as failing to associate splenic vessel occlusion with extrapancreatic extension. In contrast, its accuracy for T2 or lower stages, which rely on tumor size, was excellent, indicating proficiency in interpreting numerical values.

N-factor errors stemmed from misinterpreting non-specific lymph node findings or failing to understand the concept of regional nodes specific to pancreatic cancer. M-factor classification, however, was highly accurate, as the model reliably recognized declarative statements of distant metastasis.

The accuracy for resectability was slightly higher than for T-staging, partly because M1 status automatically renders a case unresectable. Errors in resectable cases were similar to T-staging, attributable to the misinterpretation of complex anatomy, such as mistaking neural plexus invasion for major arterial involvement.

Regarding the identification of missing information, the model successfully pointed out the absence of basic data, such as tumor size or statements on regional node status. However, it revealed a key limitation: it failed to flag the absence of a specific lymph node count when vague terms such as 'multiple' were used. More critically, for resectability, while it could identify the need to clarify tumor-vessel contact, it failed to recognize the omission of the degree of circumferential involvement - a crucial detail for surgical planning. This highlights a gap in the model's understanding of quantitative assessment concepts in radiology.

Our findings align with other studies [13,16] in that providing LLMs with clear rules is effective, but they struggle with nuances that require expert domain knowledge. The limitations in interpreting vascular invasion may impact the determination of surgical eligibility, suggesting a need for further improvement for future clinical application. An LLM may misapply guidelines if it misreads subtle phrasing in a report, underscoring the need for more standardized, 'AI-friendly' reporting language to bridge this gap.

This study has several limitations. First, the modest sample size from a single institution and a dataset skewed towards advanced tumors may limit generalizability and inflate some performance metrics. Second, our evaluation was based on the 8th edition of the Japanese General Rules for the Study of Pancreatic Cancer; therefore, the model's performance may not be directly applicable to assessments based on other international standards, such as the NCCN guidelines. Third, our reference standard was expert consensus on reports, not pathological staging for all cases. As the objective of this study was to evaluate the LLM's ability to recognize and extract necessary information for staging from the reports, we selected staging based solely on the reports as the reference standard consistent with this objective. Fourth, we evaluated a single LLM without head-to-head comparisons or exhaustive prompt engineering. Finally, our study was limited to text extraction and did not incorporate imaging data, nor does it address errors of omission in the original reports.

In summary, Claude 3.7 Sonnet demonstrates the feasibility of effectively structuring complex free-text data for pancreatic cancer staging. While not yet a replacement for expert interpretation, they show significant promise for building LLM-driven agentic workflows [17]. These workflows could assist in clinical practice by not only auto-populating draft reports or checklists for human review, but also by automatically citing and applying clinical guidelines, or suggesting subsequent necessary examinations and treatments. This semi-automation can save time, reduce documentation errors, and ultimately support clinical decision-making by presenting critical information in a clearer, more structured format. However, given the inherent limitations in the accuracy of LLMs, rigorous evaluation and appropriate supervision by radiologists are essential for their responsible implementation.

## Conclusions

Using a large language model (Claude 3.7), we successfully extracted TNM stage and resectability status from unstructured Japanese radiology reports of pancreatic cancer with good accuracy. The LLM’s performance was strongest for nodal and metastasis detection and in refraining from guessing missing information, and it showed competent (though lesser) performance for the nuanced tumor extent and resectability classifications. These results demonstrate the feasibility of zero-shot LLMs to aid in generating structured staging data from routine reports, which could facilitate data-driven care without requiring radiologists to adopt strict templates. With further improvements - such as enhanced prompt strategies (e.g., stepwise reasoning with anatomical knowledge) or modest fine-tuning - the model’s accuracy in tricky areas such as vascular invasion could be increased to near-expert levels. Overall, our study suggests that Claude 3.7 and similar LLMs can serve as valuable tools for semi-automated cancer staging in clinical workflows, helping bridge the gap between verbose radiology narratives and the structured information needed for optimal patient management.
